# Impact of Interval to Esophagectomy After Neoadjuvant Immunochemotherapy for Locally Advanced Esophageal Squamous Cell Carcinoma: A Multicenter Retrospective Cohort Analysis

**DOI:** 10.1111/1759-7714.70019

**Published:** 2025-03-03

**Authors:** Hui Xu, Zhinuan Hong, Ye Lin, Sunkui Ke, Zhen Chen, Shuhan Xie, Dinghang Chen, Kaiming Peng, Peipei Zhang, Mingduan Chen, Ziyang Han, Jihong Lin, Shuchen Chen, Jinxin Xu, Jinbiao Xie, Mingqiang Kang

**Affiliations:** ^1^ Department of Thoracic Surgery Fujian Medical University Union Hospital Fuzhou China; ^2^ Key Laboratory of Cardio‐Thoracic Surgery(Fujian Medical University) Fujian Province University Fuzhou China; ^3^ Key Laboratory of Ministry of Education for Gastrointestinal Cancer Fujian Medical University Fuzhou China; ^4^ Fujian Key Laboratory of Tumor Microbiology Fujian Medical University Fuzhou China; ^5^ Department of Thoracic Surgery, Zhongshan Hospital of Xiamen University, School of Medicine Xiamen University Xiamen China; ^6^ Department of Cardiothoracic Surgery The Affiliated Hospital of Putian University Putian China; ^7^ Department of Cardiothoracic Surgery, Putian Pulmonary Hospital Putian China

**Keywords:** esophageal squamous cell carcinoma, interval to surgery, long‐term survival, neoadjuvant immunochemotherapy(nICT), pathological response

## Abstract

**Background:**

Neoadjuvant chemoimmunotherapy (nICT) has emerged as a novel and promising treatment model for esophageal squamous cell carcinoma (ESCC). However, the optimal interval to esophagectomy after nICT remains unclear. This study aimed to explore the impact of a prolonged interval (7–10 weeks) on short‐ and long‐term outcomes compared to the standard interval (4–6 weeks).

**Methods:**

This was a multicenter retrospective cohort analysis, including three centers. Patients were diagnosed with locally advanced ESCC (cT3‐4a or cN+) and received radical resection after at least one cycle of nICT. The primary outcomes were pathological response, disease‐free survival (DFS), and overall survival (OS). Inverse probability of treatment weighting (IPTW) was utilized to balance the baseline characteristics.

**Results:**

One hundred and seventy patients were included in the study, with 123 in the standard interval group and 47 in the prolonged interval group. After IPTW, the prolonged interval group exhibited a higher pathological complete response (pCR) than the standard group, but the difference was not statistically significant (29.5% vs. 41.5%, *p* = 0.306). Additionally, although the 3‐year DFS and OS rates were higher in the prolonged interval group, these differences did not reach statistical significance. There were no statistically significant variances observed in terms of intraoperative blood loss, surgical time, postoperative hospital stays, duration of thoracic drainage tube placement, hospital expenses, or postoperative complications.

**Conclusions:**

Patients demonstrated tolerance for esophagectomy within 4–6 weeks after nICT. Based on the present findings regarding pCR, DFS, and OS, extending the time to surgery beyond 6 weeks was found to be acceptable.

## Graphical abstract

The short‐ and long‐term outcomes of different TTS after nICT.

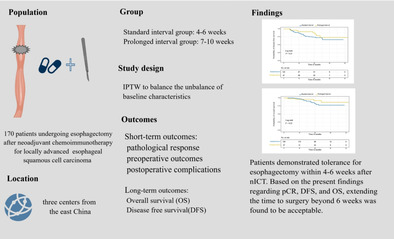

## 1 Introduction

More than 90% of esophageal cancer patients in Chinese population are diagnosed with esophageal squamous cell carcinoma (ESCC) [1]. Additionally, more than 50% of ESCC in China are locally advanced when first visit, whom require systemic therapy. Presently, the standard treatment for locally advanced ESCC is chemoradiotherapy [2]. The CROSS trial and NEOCRTEC5010 trial have confirmed the positive value of neoadjuvant chemoradiotherapy(nCRT) for ESCC [3]. However, patients still face the challenge of high recurrence and distant metastasis even though they have received standard nCRT. Thus, more novel and multimodal treatments are necessary to improve the long‐term survival of the ESCC [4]. Combination of immune checkpoint inhibitors represented by anti‐PD‐1 antibodies with chemotherapy could significantly prolong the overall survival (OS) in advanced ESCC [5].

Combination of chemotherapy and immunotherapy show a promising synergistic effect. Chemotherapy selectively depletes bone marrow immunosuppressive cells and induces immunogenic tumor cell death and lymphopenia, which could make room for the effector T cell proliferation [6]. CheckMate577 trial confirmed the positive value of adjuvant immunotherapy in patients receiving esophageal resection [7]. Then, thoracic surgeons explored that the application of neoadjuvant immunochemotherapy (nICT) in locally advanced ESCC patients could get a promising pathologic complete response (pCR) [8].

The nICT is a novel and promising neoadjuvant therapy model for the locally advanced ESCC. At present, the interval to esophagectomy after nICT was recommended as 4–6 weeks. Does the extension of the time interval between surgery and nICT would bring more pCR and better long‐term survival? There was still few research focused on this issue [9]. Thus, we designed a multicenter retrospective cohort study to explore whether the interval would affect the short‐term outcomes and prognosis among patients with advanced ESCC.

## 2 Materials and Methods

### 2.1 Study Design and Patient Selection

The study included patients meeting the following inclusion criteria: (1) diagnosis of locally advanced ESCC (cT3‐4a or cN+) at three enrollment centers from January 2019 to December 2022; (2) receipt of at least one cycle of neoadjuvant chemotherapy (nICT); (3) undergoing radical resection of esophageal cancer; and (4) providing complete clinical data. Exclusion criteria included: (1) a history of other malignant tumors within the past 5 years; (2) diagnosis of gastroesophageal borderline tumor/cardiac cancer; (3) refusal to participate in the study; and (4) undergoing only exploratory surgery or jejunostomy. This was a multicenter retrospective cohort study approved by the institutional ethics committee (2023KY028) and registered in the Chinese Clinical Trials Registry (ChiCTR2300069606). Detailed research plans can be seen on the Chinese clinical trial registry website (https://www.chictr.org.cn/showproj.aspx?proj=190649).

This work was reported in line with the STROCSS criteria [10].

### 2.2 Outcomes

The main outcomes were pathological response, DFS, and OS, while secondary outcomes included perioperative recovery metrics, postoperative complications, and hospitalization costs. The pathological response was assessed using pCR, which is defined as the absence of primary tumor and lymphatic nodular cells. Clinical and pathological stages were determined according to the 8th edition of the American Joint Committee on Cancer/International Union Against Cancer (AJCC/UICC) guidelines.

### 2.3 Treatment Plan

Pretreatment diagnosis and clinical staging were conducted using gastroscopy, enhanced cervical chest and upper abdominal computerized tomography (CT), and cervical color ultrasound, with positron emission computed tomography (PET‐CT) scans performed when necessary. PD‐1 monoclonal antibodies using in this study included camrelizumab, pembrolizumab, sintiliumab, tislelizumab, and toripalimab, administered at a dose of 200/240 mg. The main chemotherapy regimens consisted of platinum‐paclitaxel or platinum‐docetaxel, administered once every 3 weeks with doses adjusted based on patient tolerance. Contrast‐enhanced CT scans of the neck, chest, and upper abdomen were performed 3–4 weeks after the second or third neoadjuvant cycle to evaluate the neoadjuvant therapy's effectiveness. All patients underwent minimally invasive esophagotomy (MIE), involving routine secondary thoracoabdominal lymph node dissection and 3.0–3.5 cm wide gastroesophageal tube replacement. For patients with suspected cervical lymph node metastasis before surgery, three‐field lymphadenectomy was performed. Jejunostomy was routinely conducted.

### 2.4 Statistical Analysis

The operative interval, defined as the duration between the last neoadjuvant treatment and surgery, categorized patients into a standard interval group (4–6 weeks) and a prolonged group (7–10 weeks). Continuous data were presented as mean ± standard deviation, while categorical data were represented as numbers (percentage). Continuous variables were analyzed using the Mann–Whitney *U* test or Student's *t* test, while categorical variables were analyzed using the chi‐square test or Fisher exact test. Kaplan–Meier (KM) curves were employed to compare OS and DFS between the two groups.

To ensure baseline data comparability between the two groups, inverse probability of treatment weighting (IPTW) was employed to mitigate bias and influence of confounding variables. A bilateral *p*‐value < 0.05 indicated a statistically significant difference. Statistical analysis was performed using R software 4.3.1.

## 3 Results

### 3.1 Comparisons of Baseline Characteristics Before and After IPTW

**FIGURE 1:**
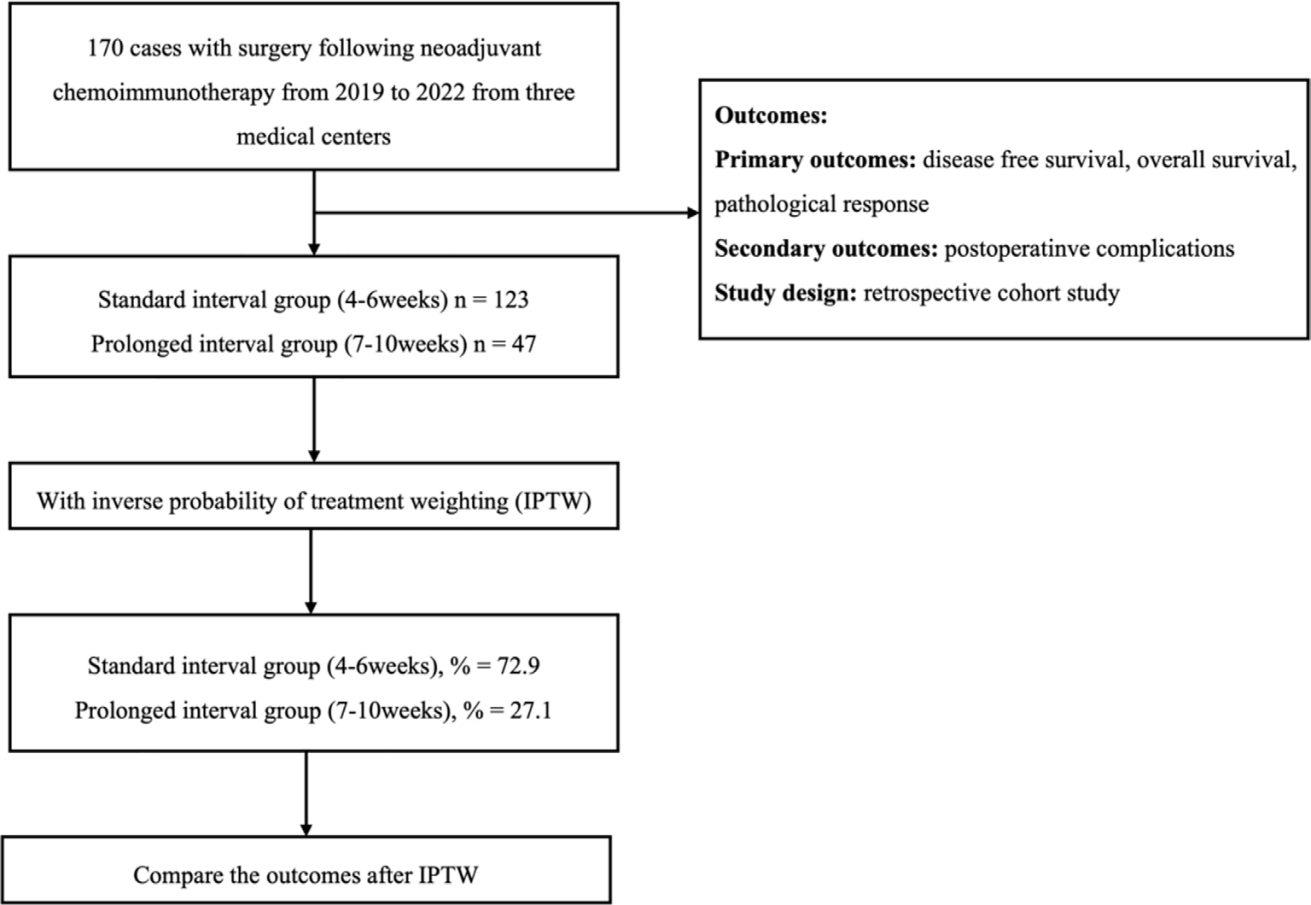
The flowchart of patient selection and analysis.

**FIGURE 2:**
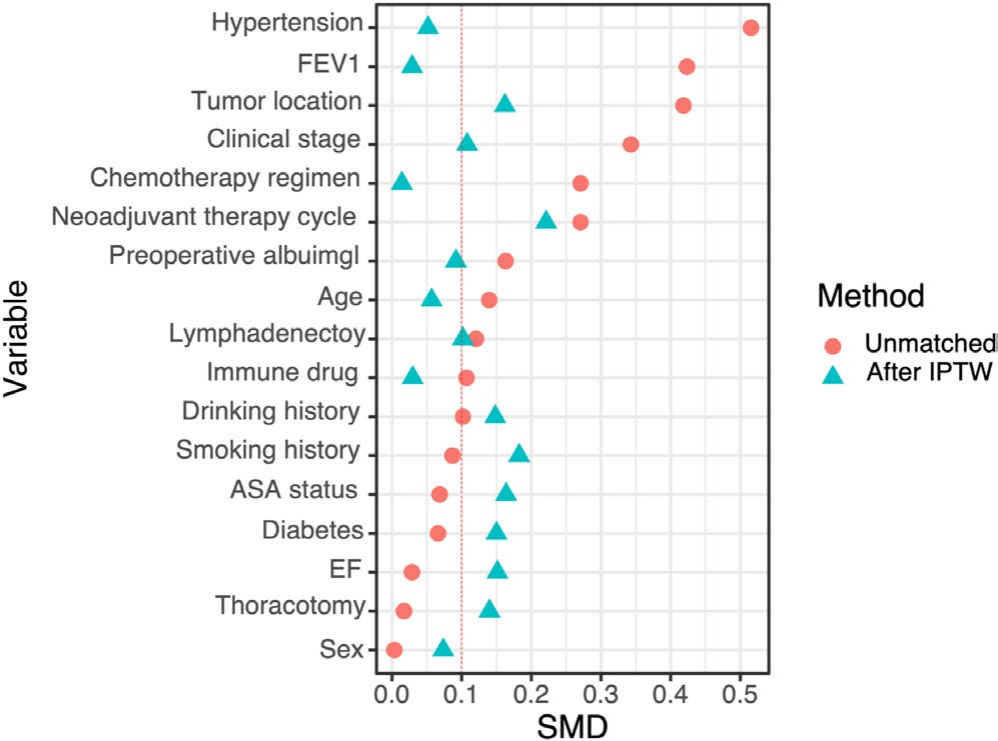
The standard mean differences of baselines characteristics before and after inverse probability of treatment weighting (IPTW).

A cohort of 170 patients from three institutions in China, spanning 2019–2022, were included. Total 123 patients received a the standard interval, and other 47 cases had a prolonged interval. Before matching, the prolonged interval group showed a lower incidence of hypertension (24.4% vs. 6.4%, *p* = 0.008) and lower mean forced expiratory volume in 1 s (FEV1) (2.75 vs. 2.51, *p* = 0.014). Following IPTW, both groups exhibited similar FEV1 means (2.68 vs. 2.69, *p* = 0.884) and hypertension prevalence (80.7% vs. 78.6%, *p* = 0.863). Clinical Stage 3 predominated in both groups (57.7% vs. 53.5%, *p* = 0.879). Patient selection and analysis process are depicted in Figure 1. Baseline characteristics between the two groups were comparable after IPTW. The *p*‐value of baseline characteristics between the two groups before and after IPTW was summarized in Table 1. The standard mean differences of baseline characteristics before and after IPTW are shown in Figure 2.

**TABLE 1:** The comparisons of baseline characteristics between the standard interval group and prolonged interval group before and after inverse probability of treatment weighting.

Characteristics	Unweighted study population, no. (%)		*p*	Weighted study population, %		*p*
	Standard group (*N* = 123)	Prolonged group (*N* = 47)		Standard Group	Prolonged Group	
Hypertension			0.008			0.863
No	93 (75.6)	44 (93.6)		80.7	78.6	
Yes	30 (24.4)	3 (6.4)		19.3	21.4	
Clinical stage			0.128			0.879
2	35 (28.5)	17 (36.2)		32.1	33.2	
3	77 (62.6)	22 (46.8)		57.7	53.5	
4a	11 (8.9)	8 (17.0)		10.2	13.3	
Sex			1.000			0.752
Female	26 (21.1)	10 (21.3)		22.6	19.6	
Male	97 (78.9)	37 (78.7)		77.4	80.4	
PD‐1 drugs			0.680			0.877
Domestic	102 (83.0)	37 (78.7)		82.9	84.0	
Pembrolizumab	21 (17.0)	10 (21.3)		17.1	16.0	
Neoadjuvant treatment cycles			0.160			0.443
≦3	113 (91.9)	39 (83.0)		85.9	77.4	
> 3	10 (8.1)	8 (17.0)		14.1	22.6	
Chemotherapy regimen			0.160			0.940
PF	113 (91.9)	39 (83.0)		88.8	89.2	
Others	10 (8.1)	8 (17.0)		11.2	10.8	
Lymphadenectomy			0.620			0.609
Two field	105 (85.4)	42 (89.4)		86.9	91.2	
Three field	18 (14.6)	5 (10.6)		13.1	9.8	
Diabetes			1.000			0.285
No	116 (94.3)	45 (95.7)		95.4	98.0	
Yes	7 (5.7)	2 (4.3)		4.6	2.0	
Drinking history			0.692			0.468
No	86 (70.0)	35 (74.5)		71.5	77.9	
Yes	37 (30.0)	12 (25.5)		28.5	22.1	
Smoking history			0.740			0.416
No	55 (44.7)	19 (40.4)		46.3	55.4	
Yes	68 (55.3)	28 (59.6)		53.7	44.6	
ASA status			0.893			0.283
2	110 (89.4)	41 (87.2)		89.7	94.2	
3	13 (10.6)	6 (12.8)		10.3	5.8	
Tumor location			0.077			0.780
Upper	17 (13.8)	2 (4.3)		11.0	6.6	
Middle	65 (52.9)	22 (46.81)		51.0	51.7	
Lower	41 (33.3)	23 (48.94)		38.0	41.7	
Thoracotomy			1.000			0.394
Video‐assisted	108 (87.8)	41 (87.2)		88.6	92.7	
Robotic‐assisted	15 (12.2)	6 (12.8)		11.4	7.3	
Age, mean (± SD), years	61.7 ± 7.3	60.7 ± 6.5	0.429	61.4 ± 7.0	61.8 ± 6.6	0.793
Body mass index			0.743			0.674
< 18.5	12 (9.8)	4 (8.5)		9.2	9.3	
18.5–24	90 (73.2)	37 (78.7)		76.3	81.6	
> 24	21 (17.1)	6 (12.8)		14.5	9.2	
Preoperative albumin (g/L), mean (± SD)	41.4 ± 3.5	41.4 ± 3.5	0.344	41.6 ± 3.5	41.9 ± 3.4	0.666
Actual FEV1, mean (± SD)	2.75 ± 0.6	2.51 ± 0.6	0.014	2.68 ± 0.6	2.69 ± 0.6	0.884
EF, mean (±SD)	67.6 ± 5.1	67.6 ± 5.2	0.866	67.6 ± 5.1	68.4 ± 5.3	0.577

Abbreviations: EF, ejection fraction; FEV1, forced expiratory volume in 1 s.

### 3.2 Comparison of the Pathological Response, DFS, and OS After IPTW

**FIGURE 3:**
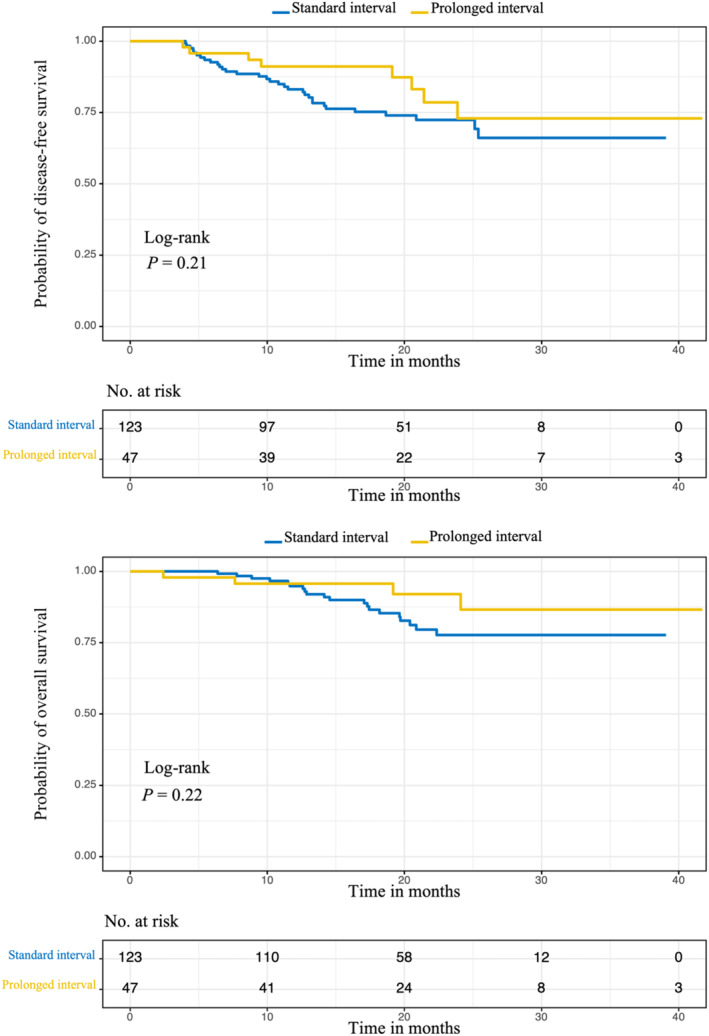
(A) The disease free survival between prolonged and standard interval group after IPTW. (B) The overall survival between prolonged and standard interval group after IPTW. Patients demonstrated tolerance for esophagectomy within 4–6 weeks after nICT. Based on the present findings regarding pathological complete response (pCR), DFS, and OS, extending the time to surgery beyond 6 weeks was found to be acceptable. The prolonged interval group exhibited a higher pCR than the standard group, but the difference was not statistically significant (29.5% vs. 41.5%, *p* = 0.306). Additionally, although the 3‐year DFS and OS rates were higher in the prolonged interval group, these differences did not reach statistical significance.

After applying IPTW, both groups demonstrated similar results in terms of the ypTNM stage. Additionally, there were no significant statistical differences between the two groups regarding pCR in the tumor (29.5% vs. 41.5%, *p* = 0.306), pCR in the tumor and lymph nodes (20.1% vs. 22.3%, *p* = 0.819), MPR (47.2% vs. 64.2%, *p* = 0.104), and tumor regression grade (TRG = 3: 27.2% vs. 31.1%, *p* = 0.671) (Table 2). Figure 3 illustrates the KM curves for DFS and OS of the total cohort after IPTW. At the time of data cutoff, neither group reached the median DFS and OS. The 3‐year DFS rates were 66.1% versus 77.1%, and the 3‐year OS rates were 76.6% versus 89.0%, with no significant difference observed in DFS (*p* = 0.21) and OS (*p* = 0.22).

**TABLE 2:** The comparisons of preoperative outcomes and pathological response between the standard interval group and prolonged interval group after inverse probability of treatment weighting.

Outcomes	Weighted study population, %		
	Standard group	Prolonged group	*p*
ypTNM stage			0.501
1	33.5	48.1	
2	15.8	7.9	
3a	19.4	14.4	
3b	30.1	26.5	
4a	1.3	3.1	
pCR in tumor			0.306
No	70.5	58.5	
Yes	29.5	41.5	
pCR in tumor and LN			0.819
No	79.9	77.7	
Yes	20.1	22.3	
MPR			0.104
No	52.8	35.8	
Yes	47.2	64.2	
Pathological grade			0.071
0	27.8	41.5	
1	19.4	22.7	
2	25.0	3.6	
3	27.2	31.1	
4	0.6	1.2	
Poor pathological response (TRG = 3)			0.671
No	72.8	68.9	
Yes	27.2	31.1	
Surgical time (min), median [IQR]	340.1 [295.0, 375.0]	347.0 [294.0, 391.0]	0.689
Intraoperative blood loss(mL), median [IQR]	134.0 [100.0, 200.0]	172.7 [100.0, 200.0]	0.136
Thoracic drainage tube stays (days), median [IQR]	11.1 [7.0, 12.0]	11.5 [8.00, 14.0]	0.777
Hospital stays (days), median [IQR]	21.7 [13.0, 25.0]	21.4 [14.0, 29.0]	0.860
Postoperative hospital stays (days), median [IQR]	15.7 [9.0, 17.0]	14.9 [12.0, 18.0]	0.582
Hospital expenses (RMB), median [IQR]	94 982.9 [76 979.6, 102 023.7]	89 224.3 [78 742.8, 102 536.1]	0.332

Abbreviations: MPR, major pathological response; pCR, pathological complete response; RMB, Renminbi, the official currency of the People's Republic of China.

### 3.3 Comparisons of Preoperative Outcomes and Postoperative Complications After IPTW

As presented in Table 2, both groups had comparable surgical times (340.1 vs. 347.0 min, *p* = 0.689) and intraoperative blood loss (134.0 vs. 172.7 mL, *p* = 0.136), as well as similar durations of thoracic drainage tube placement (11.1 vs. 11.5 days, *p* = 0.777). Additionally, both groups had similar postoperative hospital stays (15.7 vs. 14.9 days, *p* = 0.860) and hospital expenses (94982.9 vs. 89224.3, *p* = 0.332).

The postoperative complication rates were comparable between the two groups, with rates of postoperative pneumonia (27.9% vs. 27.6%, *p* = 0.975) and anastomotic leakage (10.3% vs. 10.4%, *p* = 0.994). Furthermore, both groups exhibited similar rates of other postoperative complications. The comparisons of postoperative complications between the standard interval group and the prolonged interval group after IPTW are presented in Table 3.

**TABLE 3:** The comparisons of postoperative complications between the standard interval group and prolonged interval group after inverse probability of treatment weighting.

Outcomes	Weighted study population, %		
	Standard group	Prolonged group	*p*
Pneumonia			0.975
No	72.1	72.4	
Yes	27.9	27.6	
Anatomic leakage			0.994
No	89.7	89.6	
Yes	10.3	10.4	
Palsy of recurrent laryngeal nerve			0.081
No	97.2	85.9	
Yes	2.8	14.1	
Cardiac events			0.908
No	82.3	83.3	
Yes	17.7	16.7	
Chylothorax			0.618
No	97.9	98.8	
Yes	2.1	1.2	
Bleeding			0.780
No	97.2	96.5	
Yes	2.8	3.5	
Pleural effusion			0.451
No	68.5	60.8	
Yes	31.5	39.1	
Vomiting			0.929
No	97.3	97.6	
Yes	2.7	2.4	
Analgesic			0.305
No	64.3	52.6	
Yes	35.7	47.4	
Electrolyte disturbance			0.534
No	69.2	74.8	
Yes	30.8	25.2	

## 4 Discussion

The treatment of locally advanced ESCC is still a great challenge for clinicians [11, 12]. Esophagectomy after nCT or nCRT has been shown to improve long‐term survival [13, 14, 15]. However, even patients receiving high‐intensity treatment still have a poor prognosis [16]. The value of immunotherapy as an adjuvant therapy for esophageal cancer has been confirmed in previous studies [17]. Further, many Phase II clinical trials have confirmed the safety and effectiveness of nICT mode for locally advanced ESCC [18, 19, 20, 21]. It is necessary to study the interval after nICT for short‐term and long‐term outcomes to promote this model.

When we considering this issue, we should take both the tolerance of surgery and the oncology benefit into consideration. The time to surgery (TTS) after neoadjuvant therapy has been studied in other types of tumors, such as rectal cancer [20, 22]. The interval between the nCRT and surgery was considered as an important factor in predicting pCR rate. Extending the TTS after the neoadjuvant therapy has been shown to increase the likelihood of tumor degradation (pCR from 10% to 26%) without affecting postoperative complications, OS, and PFS [23]. Shapiro found that extending TTS after nCRT could increase the pCR rate, which, in turn, could improve prognostic outcomes [24]. However, there was controversy about whether prolonged TTS after nCT or nCRT affects patients' outcomes and postoperative complications [25, 26].

Most studies have reported that prolonged TTS can improve the pCR rate. Shaikh retrospectively analyzed 88 patients with esophageal cancer and found that the pCR rate increased from 12.5% to 40.9% when the TTS was extended to 45 days [27]. A study led by Haisley found that patients had the highest pCR rate when the TTS between the neoadjuvant chemotherapy and esophageal surgery was 85–98 days [28]. Ranney DN observed that patients with longer TTS (≥ 8 weeks) after nCRT had a better pathologic downstaging, but had worse long‐term outcomes [29]. Similarly, Qin found that TTS longer than 7–8 weeks significantly increased the pCR rate compared with shorter TTS but also showed a poorer 2‐year OS and 5‐year OS [25]. However, until now, there have been few studies exploring the impact of TTS after nICT in advanced esophageal cancer. In this study, we found that the prolonged group tended to have a better pCR rate, although the difference was not statistically significant (29.5% vs. 41.5%, *p* = 0.306, after IPTW). Furthermore, although the 3‐year DFS and OS rates were higher in the prolonged interval group, the differences were not statistically significant. This lack of significance may be attributed to the small sample size. Larger samples and randomized controlled trials are needed to confirm these findings.

Postoperative complications and surgical mortality increased when surgery was delayed for more than 7–8 weeks after nCT [25]. Patients typically require about 4 weeks to recover after receiving nCT [23]. Delaying the TTS beyond 8 weeks can lead to significant tissue fibrosis, increasing surgical difficulty, and the risk of ongoing tumor growth and metastasis [30]. Wang et al. found that a shorter TTS (less than 21 days) reduced the tumor‐positive margin rate and surgical mortality, while also increasing the R0 resection rate. Conversely, a TTS more than 12 weeks was associated with poorer OS due to a lower R0 resection rate and higher surgical mortality [31]. Wakita et al. reported that an extended interval after nCRT would increase the risk of postoperative complications such as anastomotic leakage and recurrent laryngeal nerve palsy [32], with Nilsson et al. [26] reporting similar findings. In this study, we found no significant difference in the operation time, intraoperative blood loss, postoperative hospital stays, chest drainage time, postoperative complications, or hospital expenses between the two groups, which indicates that patients could tolerate the trauma of esophagectomy after a 3‐week interval. Present evidence also suggests that a prolonged interval does not increase tissue fibrosis and surgical difficulty.

## 5 Limitations

We involved patients from three centers and employed strict inclusion criteria to ensure the robustness of our study. However, this research has several notable limitations. First, being a retrospective study, it was inherently challenging to adequately control for all potential confounders, which might have impacted the results. Second, the follow‐up period was relatively short, limiting our ability to draw conclusions beyond the midterm prognosis. To fully understand the impact on long‐term survival, a extended follow‐up is necessary. Third, the relatively small sample size may have restricted our statistical power, potentially affecting the generalizability of our findings. Therefore, our findings require validation through larger‐scale randomized controlled trials.

## 6 Conclusions

There were no significant differences in perioperative recovery, intraoperative conditions, or postoperative complications between the standard interval group and the prolonged interval group, indicating that patients could tolerate surgery 4–6 weeks after nICT. While DFS and OS appeared better in the prolonged interval group, further validation is needed. Currently, there is no clear evidence suggesting that extending TTS beyond 6 weeks negatively impacts postoperative complications and prognosis.

## Author Contributions

All authors contributed to the conception and design of the study. Hui Xu, Zhinuan Hong and Jinxin Xu contributed to the study design. Hui Xu and Zhinuan Hong contributed to the drafting of the article. Ye Lin, Dinghang Chen and Zhen Chen contributed to data collection and imaging analysis. Hui Xu and Shuhan Tie participated in data analysis and interpretation and led the revision of the article. Mingqiang Kang, Shuchen Chen, Jinbiao Xe supervised the study. All authors reviewed and approved the final manuscript.

## Conflicts of Interest

The authors declare no conflicts of interest.

## Data Availability

Data that support the findings of this manuscript is available from the corresponding author, upon reasonable request.
